# 4.11 A/1650 V Sapphire-Substrate GaN MIS-HEMTs with Thin Buffer for Medium-Voltage Power Applications

**DOI:** 10.3390/mi17020233

**Published:** 2026-02-11

**Authors:** Changhao Chen, Yang Liu, Xiaowei Zhou, Peixian Li, Yongfeng Zhang, Bo Yang, Zili Yang, Junchun Bai

**Affiliations:** 1 School of Advanced Materials and Nanotechnology, Xidian University, Xi’an 710071, China; 23141214048@stu.xidian.edu.cn (C.C.);; 2 State Key Discipline Laboratory of Wide Band Gap Semiconductor Technology, Xidian University, Xi’an 710071, China; 3 Shanghai Gejing Semiconductor Co., Ltd., Shanghai 200120, China

**Keywords:** sapphire substrates, GaN MIS-HEMTs, thin buffer layer, high-temperature operation, long-term reliability

## Abstract

The substantially lower breakdown electric field of Si compared to GaN necessitates thick buffer layers in Si-based GaN power devices for medium-voltage applications, significantly increasing cost. Recently, sapphire substrates, offering high electrical insulation and excellent mechanical strength, have emerged as a promising alternative. In this work, we demonstrate a CMOS-compatible process for sapphire-based GaN MIS-HEMTs utilizing a thin buffer layer. The fabricated devices with a W_G_ of 20.4 mm and an L_GD_ of 24 μm achieve a high off-state breakdown voltage >1650 V and a maximum on-state current > 4.1 A, with tight statistical distributions of V_TH_ and R_ON_ across the wafer. Furthermore, statistical characterization results of dynamic R_ON_ and leakage current under electrical stress conditions at both room temperature and 150 °C, confirm operational viability at high temperatures. Finally, long-term reliability for 650 V operation is validated by high-temperature reverse bias (HTRB) accelerated aging tests.

## 1. Introduction

Gallium Nitride (GaN), as a representative wide bandgap (3.4 eV) semiconductor material, exhibits outstanding physical properties, including high electron saturation velocity (2.7 × 10^7^ cm/s), high breakdown electric field (3.3 MV/cm), and the capability for high-temperature operation. In recent years, lateral GaN power devices (GaN high-electron-mobility transistors, HEMTs), leveraging the high-density (>1 × 10^13^ cm^−2^), high-mobility two-dimensional electron gas (2DEG) (with mobility >1500 cm^2^/(V·s) at room temperature) induced by polarization effects at the AlGaN/GaN heterojunction interface, have demonstrated significant potential for high-power switching systems and have been rapidly adopted in applications operating at voltages below 650 V, such as power supplies, data centers, and consumer electronics [1,2,3,4].

Currently, driven by production cost and thermal management considerations, commercial GaN power devices predominantly utilize mature large-diameter Si (111) substrates for heteroepitaxy. However, due to the significantly lower breakdown electric field of Si (0.3 MV/cm) compared to GaN, thick buffer layers are typically required to enhance the vertical breakdown voltage capability of the devices [5,6]. This requirement for such buffer layers has resulted in reduced yield and increased production costs for GaN-on-Si wafers suitable for medium-voltage power applications, severely limiting the adoption of GaN-on-Si devices in applications requiring voltages above 1200 V, such as electric vehicles and industrial systems. In contrast, sapphire substrates, while exhibiting relatively poor thermal conductivity (~0.25 W/(cm·K) at 100 °C), possess a high breakdown electric field (>5 MV/cm) and a high mechanical strength. Furthermore, benefiting from advancements in large-diameter sapphire substrate manufacturing driven by the LED industry, low-cost sapphire substrates have been extensively researched in recent years and are gradually being commercialized [7].

Recently, Li et al. reported depletion-mode (D-mode) GaN metal-insulator-semiconductor high-electron-mobility transistors (MIS-HEMTs) on 6-inch sapphire substrates utilizing a 1.5 μm thin buffer layer for 1700 V high-power applications [8]. Their study demonstrated that the insulating sapphire substrate effectively blocks vertical leakage paths through the buffer layer and significantly suppresses lateral parasitic channels at the epilayer/substrate interface. Moreover, it is well established that increasing the gate-to-drain distance (L_GD_) in lateral GaN power devices enhances their lateral breakdown voltage. Due to the high vertical breakdown capability provided by the insulating sapphire substrate, this enhancement contributes directly to improved overall device breakdown characteristics. Consequently, Li et al. subsequently extended the L_GD_ to 100 μm and successfully fabricated D-mode GaN MIS-HEMTs, achieving breakdown voltages exceeding 8 kV [9]. Furthermore, Cui et al. employed a gate termination extension structure to fabricate enhancement-mode (E-mode) p-GaN gate HEMTs on sapphire substrates. These devices, with an L_GD_ of 27 μm, demonstrated breakdown voltages exceeding 2.5 kV [10]. Later, Yu et al. from the same research group increased the L_GD_ to 77 μm, resulting in devices with breakdown voltages surpassing 9 kV [11]. These results underscore the significant advantages of sapphire substrates for achieving high breakdown voltages in medium/high-voltage lateral GaN power devices.

However, for GaN power devices operating in medium/high-voltage power switching applications, it is also necessary to consider the impact of high-temperature operating environments and elevated junction temperatures on device electrical characteristics and long-term operational reliability [12,13]. This consideration is particularly critical for sapphire-based devices, which exhibit relatively poor substrate thermal conductivity. Unfortunately, reports on the high-temperature performance of sapphire-based GaN MIS-HEMTs with a thin buffer layer are still limited, hindering an accurate assessment of their commercialization potential.

In this work, we designed and fabricated sapphire-based GaN MIS-HEMTs featuring a thin buffer layer, which achieved a room-temperature breakdown voltage exceeding 1650 V and a maximum on-state current of over 4.1 A (measured at V_GS_ = 0 V). After providing a detailed description of the device structure and fabrication process flow, we assessed the feasibility of this low-cost, CMOS-compatible GaN power device technology for commercial-scale manufacturing through statistical analysis and electrical mapping of wafer-level threshold voltage (V_TH_) and on-resistance (R_ON_) distributions. Subsequently, we presented statistical results for dynamic R_ON_ and leakage current at both room temperature and elevated temperature (150 °C). These results provide insight into the viability of these devices for high-temperature operation. Finally, we further validated long-term operational reliability under 650 V application conditions through high-temperature reverse bias (HTRB) accelerated aging testing at 150 °C with V_GS_ = −30 V and V_DS_ = 650 V for 168 h.

## 2. Materials and Methods

**Figure 1:**
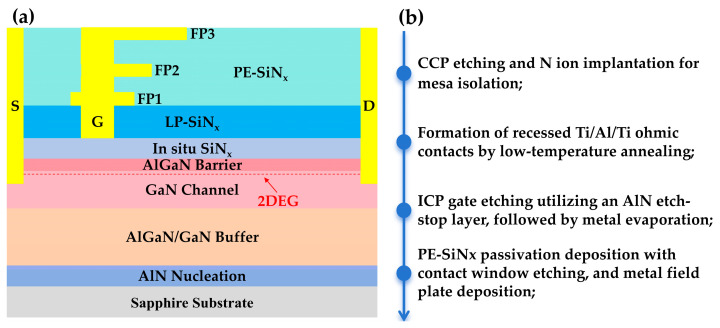
(**a**) Cross-sectional schematic of the proposed device; (**b**) Flowchart of the device fabrication process.

The AlGaN/GaN heterostructure was grown heteroepitaxially using a metal–organic chemical vapor deposition (MOCVD) system (AIXTRON CRIUS 2, Herzogenrath, Germany) on a 4-inch sapphire substrate. The epitaxial structure, depicted in Figure 1a, comprises: a 40 nm AlN nucleation layer; a 1.3 μm buffer layer consisting of a 300 nm AlGaN transition layer and a 1 μm GaN layer; a 300 nm GaN channel layer; a 20 nm AlGaN barrier layer with an Al composition of 23%; a 45 nm in situ SiN_x_ passivation layer; a 10 nm in situ AlN etch-stop layer; and a 140 nm ex situ SiN_x_ secondary passivation layer deposited by low-pressure chemical vapor deposition (LPCVD). For the 45 nm in situ SiN_x_ passivation layer, deposition was carried out using silane (SiH_4_) and ammonia (NH_3_) as the reactant gases at a chamber pressure of 120 mTorr and a substrate temperature of 1150 °C [14].

**Figure 2:**
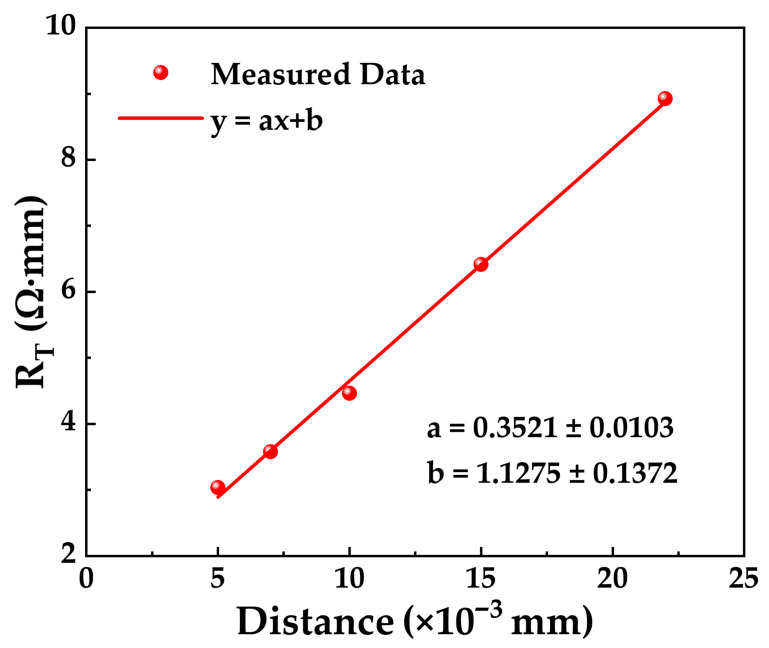
Linear fit of total resistance (R_T_) as a function of distance.

The primary process steps for device fabrication are shown in Figure 1b. Active region isolation between devices was achieved through capacitively coupled plasma (CCP) etching and nitrogen (N) ion implantation. Subsequently, a CMOS-compatible Au-free ohmic contact process with low-temperature annealing of a Ti/Al/Ti stack was carried out. First, ohmic windows were opened by inductively coupled plasma (ICP) etching, where SiN_x_ was etched with SF_6_ plasma and III-nitride was etched with BCl_3_ plasma, etching through the AlGaN layer and reaching a depth of 70 nm into the underlying GaN layer. Then, a Ti/Al/Ti (40/200/30 nm) ohmic metal stack was deposited by electron-beam (e-beam) evaporation, followed by rapid thermal annealing (RTA) at 550 °C for 120 s in a N_2_ ambient. After completing this process step, the ohmic contact resistance (R_C_) and the sheet resistance (R_SH_) were extracted using the transmission line model (TLM) in the process control monitor (PCM) area. The length and width of the TLM pads were both 100 μm. Figure 2 plots the total resistance (R_T_) as a function of pad distance and the corresponding linear fit. Least squares linear regression yields a slope a = 0.3521 ± 0.0103 and a *y*-axis intercept b = 1.1275 ± 0.1372. The R_SH_ and R_C_ are 352.1 ± 10.3 Ω/sq and 0.56 ± 0.07 Ω·mm, respectively [15].

**Figure 3:**
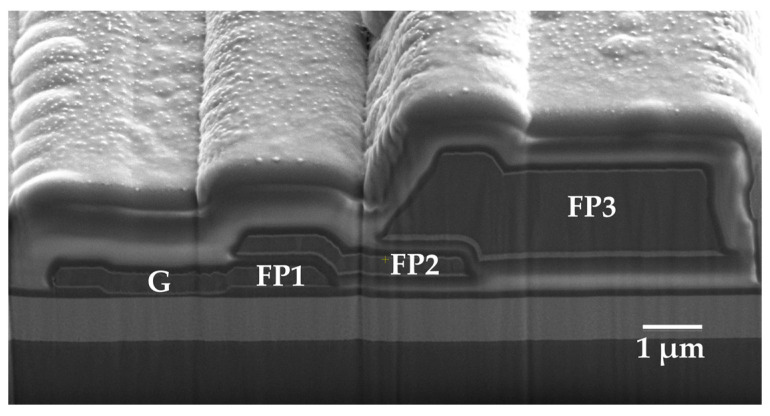
SEM image of the triple-field-plate configuration in the gate region.

The fabrication of the device gate and first field plate (FP1) commenced with inductively coupled plasma (ICP) etching. To prevent over-etching and plasma damage to the underlying in situ SiN_x_ gate dielectric layer, which is critical for maintaining its thickness and integrity to ensure threshold voltage stability and gate reliability, a 10 nm AlN etch-stop layer was deposited on the SiN_x_. This AlN layer stops the ICP etch upon exposure, enabling precise depth control and protecting the underlying SiN_x_. After ICP etching defined the gate/FP1 region and exposed the AlN within this area, the exposed AlN etch-stop layer was selectively removed using a wet etch in tetramethylammonium hydroxide (TMAH, C_4_H_13_NO) solution [16]. Subsequently, the gate and FP1 metal stack (Ti/Al/Ti, 45/340/30 nm) was deposited via e-beam evaporation. The patterning and metallization sequence was then repeated sequentially to fabricate the second (FP2) and third (FP3) field plates. Prior to the fabrication of each subsequent field plate (FP2 and FP3), a plasma-enhanced chemical vapor deposition (PECVD) SiN_x_ layer was deposited as an interlayer dielectric between the existing and the next field plate (i.e., between FP1 and FP2, and between FP2 and FP3), as illustrated in Figure 3.

**Figure 4:**
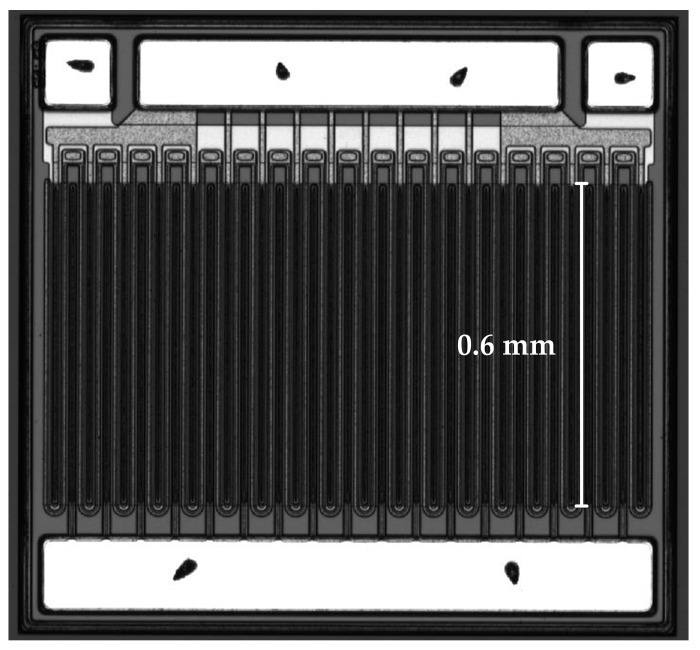
Automated Optical Inspection (AOI) image of the fabricated multi-finger gate structure, with each gate finger measuring 0.6 mm in length.

Moreover, to optimize the planar layout of large-gate-width (high-current) devices, a multi-finger gate structure was employed in the device design. As shown in Figure 4, this structure divides a single long gate into multiple shorter, parallel gate fingers (each 0.6 mm long), effectively distributing the total power dissipation while optimizing the device’s length and width dimensions [17]. Furthermore, this approach reduces performance degradation, such as current collapse, caused by localized hot spots, thus enhancing long-term reliability. Ultimately, the fabricated GaN MIS-HEMTs have a total gate width (W_G_) of 20.4 mm, a gate length (L_G_) of 2 μm, a gate-source distance (L_GS_) of 2 μm, and a gate-drain distance (L_GD_) of 24 μm.

## 3. Results

### 3.1. DC Characteristics of Devices

**Figure 5:**
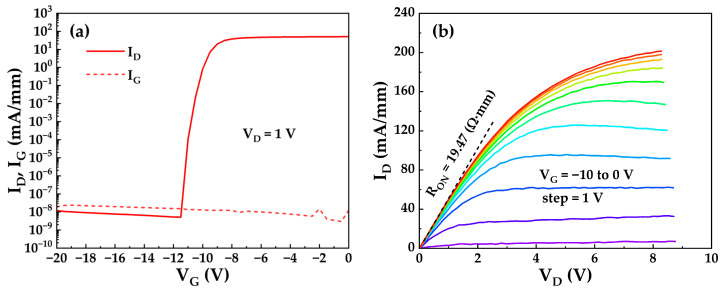
(**a**) Transfer characteristic and (**b**) output characteristic curves of a typical TO-220 packaged device.

Figure 5a presents the transfer characteristic curve of the TO-220 packaged device measured at a drain-source voltage (V_DS_) of 1 V, with the gate-source voltage (V_GS_) swept from −20 V to 0 V. The V_TH_, extracted at a constant drain current (I_D_) of 0.1 mA/mm, was −10.29 V. Benefiting from the 45 nm in situ SiN_x_ gate insulating dielectric layer, the gate leakage current (I_G_) remained below 2.5 × 10^−8^ mA/mm over the entire V_GS_ range from off-state (−20 V) to on-state (0 V), significantly reducing power dissipation during device operation. Furthermore, when the V_G_ is below −12 V, the I_G_ slightly exceeds the I_D_. This occurs because, under such gate bias conditions, the device enters the off-state where the 2DEG beneath the gate is fully depleted, interrupting the conductive path between the source and drain. At low applied V_D_ (1 V), off-state leakage mechanisms (e.g., buffer layer leakage) are considered negligible. Consequently, the low gate potential causes both source and drain currents to flow toward the gate electrode, resulting in the observed I_G_ > I_D_ characteristic. Figure 5b shows the output characteristic curves of the packaged device, with V_GS_ scanned from −10 V to 0 V in 1 V steps. The device exhibits a specific R_ON_ of 19.47 Ω·mm, corresponding to a total R_ON_ of 0.954 Ω for a gate width of 20.4 mm. Furthermore, a saturation drain current of 201.45 mA/mm was achieved at V_GS_ = 0 V and V_DS_ = 8.28 V, resulting in a total drain current of 4.11 A.

**Figure 6:**
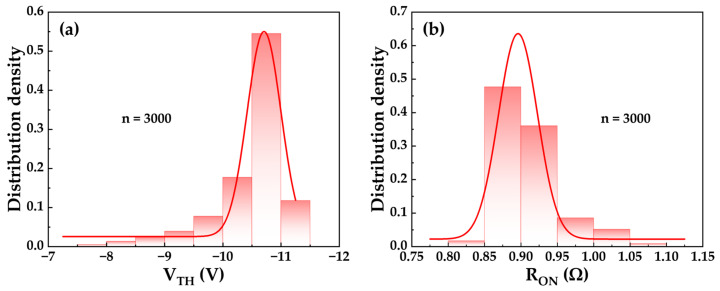
Statistical distribution across the wafer of (**a**) V_TH_ and (**b**) R_ON_ for devices with W_G_ = 20.4 mm and L_GD_ = 24 µm.

**Figure 7:**
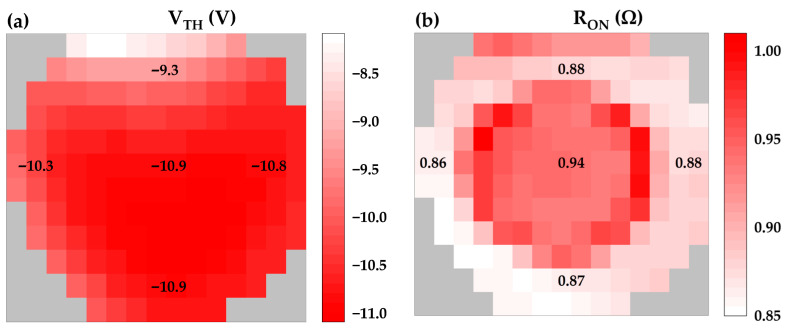
Wafer-scale electrical mapping of (**a**) V_TH_ and (**b**) R_ON_ for devices with W_G_ = 20.4 mm and L_GD_ = 24 µm.

Figure 6 presents the statistical distributions of the V_TH_ (extracted at V_DS_ = 20 V and I_DS_ = 0.1 mA) and the R_ON_ (extracted at I_DS_ = 0.1 A and V_GS_ = 0 V) for wafer-level devices obtained via full-wafer probe testing. Gaussian fitting reveals that the V_TH_ values are predominantly distributed within the range of −10.5 V to −11 V. This tight distribution highlights the precise etch depth control enabled by the AlN etch-stop layer and its effective protection of the underlying in situ SiN_x_ gate dielectric layer. The R_ON_ of the wafer-level devices was slightly lower than that of the packaged devices, attributed to the absence of the packaging process flow. The R_ON_ values are concentrated between 0.85 Ω and 0.95 Ω, demonstrating the feasibility and reproducibility of the Au-free Ti/Al/Ti low-temperature-annealed ohmic contact process implemented following material epitaxy on sapphire substrate and recess etching. Figure 7 shows the electrical mapping of V_TH_ and R_ON_ relative to their positions on the wafer, with representative parameter values annotated for five distinct regions: top, bottom, left, right, and center. Evident region-dependent characteristics are observed in the distributions of both V_TH_ and R_ON_ across the wafer. This spatial non-uniformity primarily originates from process parameter variations during epitaxial growth and device fabrication across different wafer regions. In summary, the relatively tight distributions of V_TH_ and R_ON_ across the wafer indicate the industrial viability of this CMOS-compatible, sapphire-based thin-buffer GaN MIS-HEMTs fabrication process for large-scale mass production.

### 3.2. Dynamic R_ON_ at Room Temperature and 150 °C

**Figure 8:**
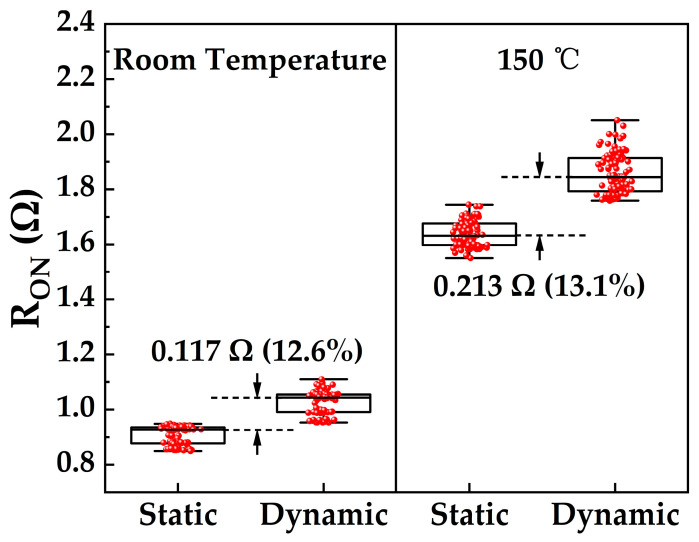
Statistical distributions of R_ON,static_ and R_ON,dyn_ for Sample A (measured at room temperature) and Sample B (measured at 150 °C).

High junction temperatures during high-power device operation and elevated-temperature application environments adversely affect device performance. Simultaneously, devices in high-power switching applications inevitably endure repeated high-electric-field and high-current stress, leading to significant current collapse. Theoretically, current collapse fundamentally arises from the trapping and release of carriers by material traps [18,19]. To evaluate the dynamic trapping behavior, two wafers were fabricated using identical processes. From each wafer, 100 devices were selected from five spatially distributed regions (center, top, bottom, left, right), with 20 devices per region. Devices from one wafer constituted Sample A, while devices from the corresponding locations on the other wafer constituted Sample B. For Sample A, static on-resistance (R_ON,static_, extracted at I_DS_ = 0.1 A and V_GS_ = 0 V) was first measured at room temperature. Subsequently, a high off-state drain bias stress (V_GS_ = −30 V, V_DS_ = 850 V, 20 ms duration) was applied three times consecutively. Dynamic on-resistance (R_ON,dyn_) was then measured immediately after gate turn-on. For Sample B, devices underwent the identical high off-state drain bias stress and subsequent R_ON,dyn_ measurement procedure, but this testing was conducted at 150 °C following a 30 min thermal stabilization period. As quantified in Figure 8, compared to R_ON,static_, R_ON,dyn_ exhibits median increases of 0.117 Ω (12.6%) at room temperature and 0.213 Ω (13.1%) at 150 °C. These relatively moderate increases in dynamic R_ON_ validate the suitability of the device fabrication process and epitaxial material quality for kilovolt-class power switching applications [20]. Furthermore, the pronounced increase in R_ON_ at 150 °C compared to room temperature is primarily attributed to enhanced optical phonon scattering resulting from intensified lattice thermal vibrations. This enhanced scattering shortens the carrier mean free path, thereby reducing carrier mobility within the device [21].

### 3.3. Leakage Current at Room Temperature and 150 °C

In power switching applications, low gate leakage current reduces power dissipation and enhances device reliability by widening the safe operating area and improving transient voltage tolerance [22,23]. Therefore, to suppress gate leakage current and extend the gate voltage swing range, a 45 nm thick in situ SiN_x_ gate dielectric layer was introduced between the gate electrode and the AlGaN barrier layer.

**Figure 9:**
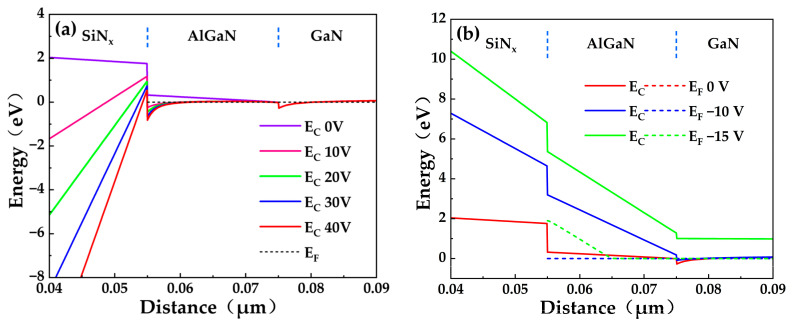
Silvaco TCAD-simulated vertical energy band diagrams under the gate showing: (**a**) downward band bending and electron accumulation under positive bias; (**b**) upward band bending and 2DEG depletion under negative bias, governing distinct gate leakage mechanisms.

To understand the gate leakage mechanisms in GaN MIS-HEMTs, we performed Silvaco technology computer-aided design (TCAD) simulations of the energy band diagram along the vertical direction under the gate across a range of gate voltages. The key models implemented in the simulation include: carrier generation/recombination models (Shockley-Read-Hall (SRH) and auger), Fermi-Dirac carrier statistics model (fermi), mobility model (albrct [24]), and polarization model specific to GaN HEMTs. As shown in Figure 9a, increasing positive gate bias induces downward band bending at the SiN_x_/AlGaN interface. When the conduction band minimum at this interface falls below the Fermi level, an electron accumulation quantum well forms. The strong vertical electric field lowers the effective AlGaN barrier height, facilitating tunneling or thermal emission of electrons from the channel 2DEG. These electrons accumulate at the SiN_x_/AlGaN interface. Owing to the high SiN_x_/AlGaN interface barrier and the relatively thick SiN_x_ gate dielectric, direct quantum mechanical tunneling of electrons is negligible at low positive gate biases. Instead, gate leakage at low positive biases occurs predominantly via trap-assisted thermal emission, governed by the temperature-dependent Poole-Frenkel Emission (PFE) mechanism, resulting in low leakage currents. Under high positive gate bias, strong electric fields induce severe band tilting within the SiN_x_ gate dielectric, forming a triangular potential barrier whose effective thickness decreases with increasing gate voltage. Concurrently, enhanced downward band bending deepens the electron accumulation well, significantly increasing both the concentration and average energy of the confined electrons. Consequently, gate leakage becomes dominated by Fowler-Nordheim Tunneling (FNT) through the triangular barrier, leading to a rapid increase in leakage current with applied gate bias [25]. Figure 9b presents the corresponding energy band diagram under negative gate bias. Increasing the magnitude of the negative gate voltage causes upward band bending in the gate region. This lifts the conduction band edge, causing the quantum well confining the 2DEG to gradually become shallower and eventually disappear. As the quantum well vanishes, the channel beneath the gate depletes, turning the device off. Under high negative bias, the depletion of the channel significantly widens the depletion region in the underlying AlGaN/GaN layer. This widened region sustains a larger fraction of the total applied gate voltage, thereby reducing the voltage drop across the SiN_x_ gate dielectric layer and lowering the electric field within the dielectric compared to the on-state. In this regime, the dominant gate leakage mechanism is again PFE [26]. PFE involves the field-assisted thermal emission of trapped electrons from defect states within the dielectric and is sensitive to defects and temperature. Thus, compared to the high positive gate bias regime (dominated by FNT), the off-state gate leakage current under high negative bias (dominated by PFE) exhibits a more gradual increase with increasing magnitude of the negative gate voltage. Furthermore, the diversion of a larger portion of the applied voltage to the widening semiconductor depletion region significantly enhances the gate breakdown voltage.

**Figure 10:**
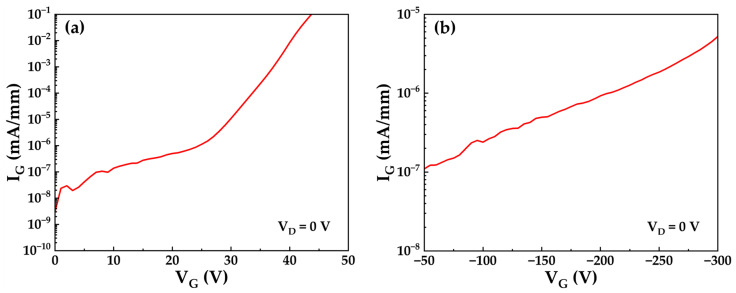
Gate leakage characteristic curves under (**a**) positive and (**b**) negative gate bias.

Figure 10 displays the gate leakage characteristic curves under positive and negative bias at room temperature. The leakage trends align with the aforementioned theoretical analysis. Using 0.1 mA/mm as the breakdown criterion, the positive gate breakdown voltage reaches 43.7 V, corresponding to a critical breakdown electric field of approximately 9.7 MV/cm within the 45 nm in situ SiN_x_ dielectric. This value falls below the theoretical limit of 14 MV/cm for SiN_x_ [27], suggesting potential for optimization in dielectric deposition processes. Under high negative bias of V_GS_ = −300 V, the gate leakage current approaches 10^−7^ mA/mm. This extremely low leakage current confirms the effectiveness of the dielectric in significantly extending the gate operating voltage range. Consequently, the gate structure reliability under high-temperature and high-power operating conditions is improved.

**Figure 11:**
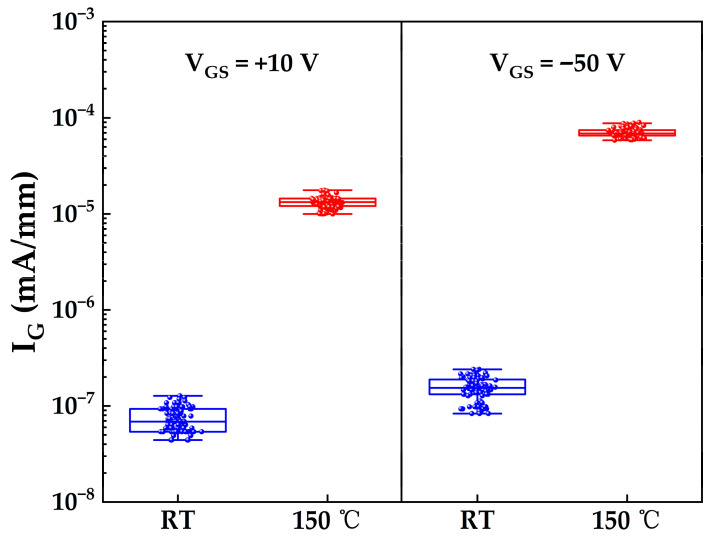
Statistical distributions of gate leakage currents under V_GS_ = +10 V and V_GS_ = −50 V for Sample A (measured at room temperature) and Sample B (measured at 150 °C).

At elevated temperatures of 150 °C, thermal energy significantly enhances the trap-assisted PFE leakage mechanism described previously, as increased thermal energy promotes the emission of trapped electrons from defect states within the bandgap into the conduction band [28]. To evaluate the impact of this mechanism on high-temperature gate leakage, gate leakage currents were measured under gate biases of +10 V and −50 V for both samples, with measurements on Sample A conducted at room temperature (RT) and on Sample B at 150 °C. As shown in Figure 11, the median gate leakage current at 150 °C is approximately two orders of magnitude higher than at room temperature under both bias conditions. Critically, even at 150 °C, the leakage currents at both +10 V and −50 V remain below the failure threshold of 1 μA/mm, demonstrating the robustness of the gate structure under high-temperature operation.

**Figure 12:**
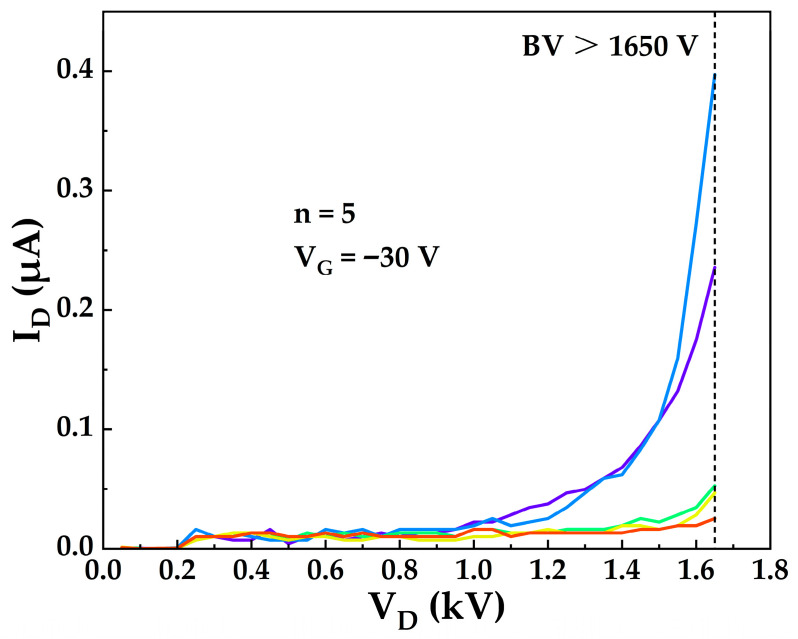
OFF-state leakage characteristic curves of 5 TO-220 packaged devices measured at room temperature.

During off-state breakdown testing at room temperature, the strong lateral electric field induced by high drain voltage extends the depletion region under the gate towards the drain side. The depletion of the 2DEG reduces the screening of the fixed positive polarization charges at the AlGaN/GaN interface. These unscreened polarization charges intensify the electric field at the drain-side gate edge, resulting in a localized high-field region. When the peak electric field in this region exceeds the critical breakdown field of the material, impact ionization occurs, triggering carrier multiplication and device avalanche breakdown. To address this issue, a triple-field-plate configuration is employed. By stacking three metal field plates with stepped extensions toward the drain terminal (extension lengths of 1.5 μm, 2 μm, and 4 μm, respectively), the localized high electric fields generated by field concentration at the edges of the gate metal and the field plates are mitigated, thus suppressing premature breakdown on the lateral device surface caused by uneven electric field distribution [8,9,29]. Figure 12 presents the off-state leakage characteristic curves of 5 TO-220 packaged devices (L_GD_ = 24 μm) measured at room temperature under a gate bias of V_GS_ = −30 V, with a maximum drain current compliance set to 10 μA. The off-state drain current remains below 0.4 μA at drain voltages up to 1650 V, demonstrating an off-state breakdown voltage exceeding 1650 V, defined at the drain current compliance level of 10 μA. This high breakdown voltage is primarily attributed to the triple-field-plate configuration suppressing the peak electric field at the drain-side gate edge, in conjunction with the insulating sapphire substrate, which prevents premature vertical breakdown and thereby enables the realization of high lateral breakdown performance.

To evaluate the off-state leakage currents at elevated temperatures, drain and gate leakage currents were measured under V_GS_ = −30 V and V_DS_ = 850 V for both samples, with measurements on Sample A conducted at room temperature and on Sample B at 150 °C.

**Figure 13:**
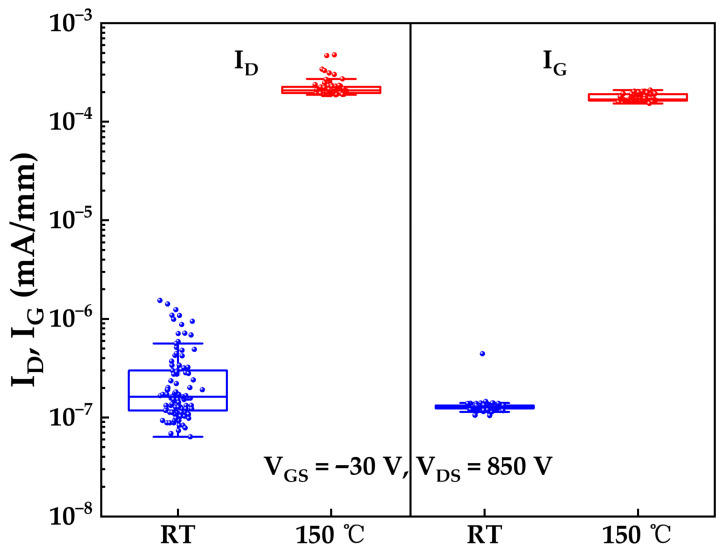
Statistical distributions of off-state leakage currents (drain current I_D_, gate current I_G_) under V_GS_ = −30 V and V_DS_ = 850 V for Sample A (measured at room temperature) and Sample B (measured at 150 °C).

A V_GS_ of −30 V was selected to both verify the reliability of the gate structure during the turn-off test and to provide a sufficient gate voltage margin for subsequent testing, ensuring the device remains fully turned-off. Separately, a V_DS_ of 850 V was chosen to establish the necessary drain voltage margin required for subsequent packaging and reliability tests, aiming to reduce potential failures during post-packaging HTRB accelerated aging tests. Additionally, voltages exceeding 850 V were deliberately avoided to prevent excessive damage or potential batch failure during this preliminary assessment, particularly at the elevated temperature of 150 °C. As shown in Figure 13, at 150 °C, both leakage currents increase significantly compared to room temperature. Notably, the drain and gate leakage currents at 150 °C exhibit comparable magnitudes, which could suggest that gate dielectric leakage constitutes a significant component of the total off-state leakage current under these bias conditions. To reduce this gate-related leakage, optimizing the gate dielectric (e.g., increasing thickness and/or improving deposition quality) could be beneficial. Furthermore, Figure 13 shows that the drain leakage current at 150 °C approaches its failure threshold of 1 μA/mm. This elevated leakage level poses a concern for practical high-power switching operation. Under sustained high-voltage stress, the flow of leakage currents can generate localized heating (hot spots). This temperature rise typically increases the intrinsic carrier concentration and leakage current, potentially establishing a positive feedback mechanism. If unchecked, this could lead to a continuous escalation of leakage current, exceeding the failure threshold, causing performance degradation, and ultimately culminating in device failure (e.g., breakdown or burnout). Therefore, given the observed high-temperature leakage characteristics and the associated thermal runaway risk, the commercial viability of these devices requires further validation through rigorous reliability testing, such as HTRB accelerated aging tests.

### 3.4. Results of HTRB Accelerated Aging Testing

As previously discussed, current collapse and leakage currents exhibited a significant increase under combined thermal and electrical stress. Therefore, to evaluate long-term reliability under high-temperature/high-voltage operation, eighty TO-220 packaged devices underwent HTRB accelerated aging testing at T = 150 °C, V_GS_ = −30 V, V_DS_ = 650 V for t = 168 h [30]. Key electrical parameters of the devices were measured at room temperature both before and after HTRB testing. For clarity, these two sets of room-temperature measurements are hereafter designated as FT1 (pre-stress) and FT2 (post-stress).

**Figure 14:**
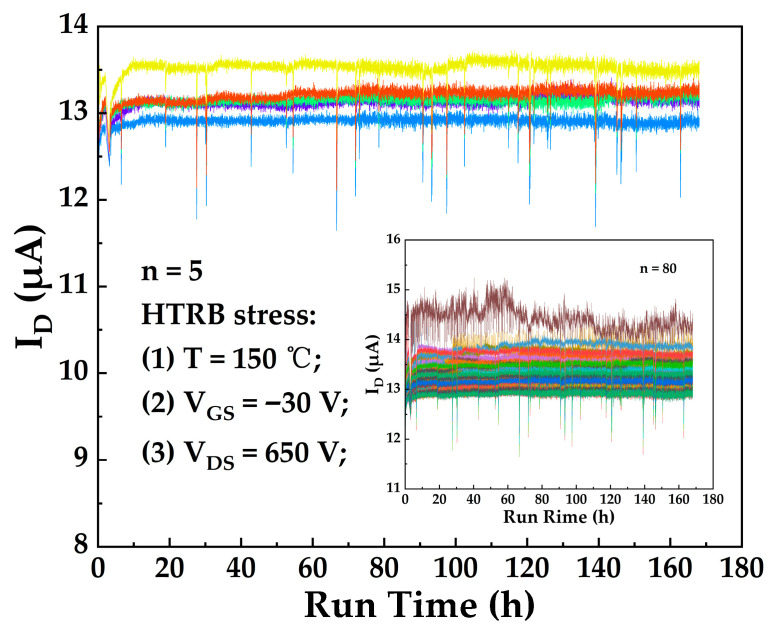
Time evolution of off-state drain current (I_D_) for five randomly selected TO-220 packaged devices under HTRB stress. The inset displays the I_D_ progression of all eighty devices.

Figure 14 displays the evolution of the off-state drain current (I_D_) over time during HTRB testing for five randomly selected TO-220 packaged devices. The inset shows the I_D_ evolution for all eighty devices. Due to the similar magnitude of leakage currents, the curves exhibit high overlap, justifying their presentation in the inset. It can be observed that I_D_ of the packaged devices stabilized in the range of 12–15 μA during HTRB stress. This microampere-range drain leakage current is attributed to the Trap-Assisted Thermionic Field Emission (TATFE) mechanism: under combined high-temperature and high-bias stress, electrons originating from both the valence band and trap-occupied states acquire sufficient energy to undergo thermionic emission via defects. These emitted electrons are subsequently swept into the channel drift region by the strong lateral electric field [31].

Median values of key electrical parameters obtained from room-temperature FT1 and FT2 tests are summarized in Table 1. The parameters and their extraction conditions are as follows: V_TH_ at V_DS_ = 20 V and I_DS_ = 0.1 mA; R_ON_ at I_DS_ = 0.1 A and V_GS_ = 0 V; I_GN_ at V_GS_ = −50 V; I_GP_ at V_GS_ = 20 V; I_D_ at V_GS_ = −30 V and V_DS_ = 650 V. Comparison of the FT1 and FT2 results indicates no significant change in device leakage current after HTRB stress. Additionally, the median R_ON_ value exhibits an increase of less than 5% relative to the pre-stress baseline. These findings provide preliminary validation of the device’s long-term operational reliability within the 650 V application platform. Moreover, a significant negative shift in the V_TH_ is observed. This shift is primarily attributed to the accumulation of uncompensated positive charges beneath the gate. These charges are generated during thermal and electrical stress via electron emission from both bulk traps within the dielectric layer and interfacial traps at the AlGaN/SiN_x_ interface, which constitutes the fundamental mechanism of negative bias/temperature instability (NBTI) [32]. Consequently, since this charge accumulation mechanism is intrinsic to MIS gate structures under stress, the resulting NBTI is inherently unavoidable in such devices, adversely impacting their operational reliability in practical implementations. Therefore, optimization of the gate dielectric layer material deposition process and assessment of the magnitude of device threshold voltage drift prior to deployment are essential, as these factors directly influence the operational lifetime of the devices in application systems. Furthermore, this NBTI phenomenon exhibits strong temperature dependence. Relevant studies report that the magnitude of the negative V_TH_ shift increases significantly with rising temperature and saturates at elevated temperatures. The specific saturation temperature is strongly dependent on the properties of the gate dielectric material and the quality of the dielectric/semiconductor interface [33].

## 4. Discussion

In fact, high gate leakage current at elevated temperatures is a critical factor limiting the pursuit of higher-voltage applications of current-processed devices. In conjunction with elevated temperature and material defect density, this leakage can trigger a degradation mechanism during prolonged operation, compromising the long-term reliability of the gate structure. Increasing the thickness and optimizing the deposition quality of the in situ SiN_x_ gate dielectric offers a direct approach to mitigate this issue [34]. However, an excessively thick gate dielectric layer degrades gate control capability, adversely impacting device switching speed and V_TH_ stability. Therefore, an inherent trade-off exists between high-voltage reliability and switching performance. Alternatively, while maintaining the gate dielectric thickness, partially replacing the in situ SiN_x_ with materials such as Al_2_O_3_ (bandgap energy E_g_~7 eV, dielectric constant ε~9), ZrO_2_ (E_g_~7.8 eV, ε~16–23), or N_2_O-treated TiO_2_ (ε~80–120 for its rutile phase) may offer a promising pathway to simultaneously reduce gate leakage and improve gate control capability [35,36,37].

While optimizing the gate dielectric is crucial for gate reliability, achieving the requisite high breakdown voltages for medium/high-voltage applications presents additional fundamental challenges. As discussed previously, achieving a substantial increase in device breakdown voltage inevitably necessitates an extension of the L_GD_. However, this approach is not entirely beneficial. In multi-finger gate configurations, a larger L_GD_ significantly increases the effective chip area, thereby escalating design complexity and manufacturing costs. Furthermore, this extended L_GD_ inherently leads to significantly increased R_ON_, which increases both switching and conduction losses under high-temperature and high-voltage operation [38]. Consequently, lateral GaN power devices face inherent disadvantages in medium/high-voltage applications. Under these constraints, AlN/AlGaN/AlN HEMTs [39,40], leveraging the enhanced breakdown electric field afforded by materials with a wider overall bandgap, represent a promising research avenue for improving the competitiveness of such devices in medium/high-voltage applications.

## 5. Conclusions

We present a CMOS-compatible fabrication process flow for low-cost sapphire-based GaN MIS-HEMTs with a thin buffer layer. Devices fabricated using this flow achieve a breakdown voltage >1650 V with a maximum on-state current >4.1 A. This process incorporates a gate-last process utilizing a 10 nm thick AlN etch-stop layer on in situ SiN_x_ and an Au-free Ti/Al/Ti low-temperature annealing ohmic contact process. The fabricated devices on the wafer demonstrate relatively concentrated statistical distributions of V_TH_ and R_ON_, which indicates feasibility for commercialization and mass production. Furthermore, statistical characterization results of dynamic R_ON_ and leakage current for the devices at both room temperature and 150 °C are reported, validating their suitability under high-temperature operation. Finally, the long-term operational reliability of the devices operating within a 650 V application platform was verified through HTRB accelerated aging tests. This low-cost fabrication process, combined with high-reliability device performance, accelerates the adoption of GaN technology in the medium-voltage market.

## Figures and Tables

**Table 1:** Median values of room temperature electrical parameters from FT1 and FT2 tests.

	V_TH_ (V)	R_ON_ (Ω)	I_GN_ (μA)	I_GP_ (μA)	I_D_ (μA)
FT1	−10.84	0.979	0.016	0.0135	0.0056
FT2	−15.43	1.022	0.015	0.0122	0.011

## Data Availability

All data that support the findings of this study are included within the article.
